# Efficacy of Oral Vaccine against Classical Swine Fever in Wild Boar and Estimation of the Disease Dynamics in the Quantitative Approach

**DOI:** 10.3390/v13020319

**Published:** 2021-02-20

**Authors:** Enkhbold Bazarragchaa, Norikazu Isoda, Taksoo Kim, Madoka Tetsuo, Satoshi Ito, Keita Matsuno, Yoshihiro Sakoda

**Affiliations:** 1 Laboratory of Microbiology, Department of Disease Control, Faculty of Veterinary Medicine, Hokkaido University, Kita 18, Nishi 9, Kita-Ku, Sapporo 060-0818, Hokkaido, Japan; bazarragchaa@vetmed.hokudai.ac.jp (E.B.); tatatataksoo-u2@eis.hokudai.ac.jp (T.K.); m.tetsuo@frontier.hokudai.ac.jp (M.T.); 2 Unit of Risk Analysis and Management, Research Center for Zoonosis Control, Hokkaido University, Kita 20, Nishi 10, Kita-Ku, Sapporo 001-0020, Hokkaido, Japan; satosito@ucm.es (S.I.); matsuk@czc.hokudai.ac.jp (K.M.); 3 International Collaboration Unit, Research Center for Zoonosis Control, Hokkaido University, Kita 20, Nishi 10, Kita-Ku, Sapporo 001-0020, Hokkaido, Japan

**Keywords:** classical swine fever, epidemiology, oral vaccine, wild boar, quantitative analysis

## Abstract

Classical swine fever virus (CSFV) in the wild boar population has been spreading in Japan, alongside outbreaks on pigs, since classical swine fever (CSF) reemerged in September 2018. The vaccination using oral bait vaccine was initially implemented in Gifu prefecture in March 2019. In the present study, antibodies against CSFV in wild boar were assessed in 1443 captured and dead wild boars in Gifu prefecture. After the implementation of oral vaccination, the increase of the proportion of seropositive animals and their titer in wild boars were confirmed. Quantitative analysis of antigen and antibodies against CSFV in wild boar implies potential disease diversity in the wild boar population. Animals with status in high virus replication (Ct < 30) and non- or low-immune response were confirmed and were sustained at a certain level after initial oral vaccination. Through continuous vaccination periods, the increase of seroprevalence among wild boar and the decrease of CSFV-positive animals were observed. The epidemiological analysis based on the quantitative virological outcomes could provide more information on the efficacy of oral vaccination and dynamics of CSF in the wild boar population, which will help to improve the implementation of control measures for CSF in countries such as Japan and neighboring countries.

## 1. Introduction

Classical swine fever (CSF) is considered one of the most devastating emergent, multisystemic viral diseases of pigs and is a notifiable disease by the World Organization for Animal Health (OIE) [1]. The disease is caused by the CSF virus (CSFV), belongs to the *Pestivirus* genus within the *Flaviviridae* family, and is closely related to other members of the genus, including bovine viral diarrhea virus genotypes 1 and 2 and border disease virus. CSFV is an enveloped virus carrying a positive-sense single-stranded RNA genome approximately 12.3 kb in length with one large open reading frame flanked by an uncapped 5’-UTR and an adenosine-uridine rich 3’-UTR region. Based on 5’-UTR and E2 encoding regions, CSFV is genetically classified into three genotypes (1, 2, and 3) and variable sub-genotypes (1.1–1.7, 2.1–2.3, and 3.1–3.4) [2]. Susceptible and reservoir hosts of CSF are swine species, including domestic pigs and wild boar.

The forms of CSFV infection in host animals are highly variable depending on the virus and host factors, such as the virulence of the strain and host immune responses, which differ by the age of the host and presence of secondary infection. The forms are classified as acute, chronic, and persistent infections [3,4]. Acute infection is caused by a highly virulent strain that causes the most severe illness with high mortality within 2–4 weeks. On the other hand, infected pigs survive over 30 days after the virus infection with moderate and disappearing clinical signs which are considered to be the chronic form of CSF [2]. Pigs with chronic infection shed the virus until they die, and specific antibodies will be detected without elimination of the virus. Persistent infection is usually caused by CSFV strains with moderate or low virulence, and this infection form is developed on the response of the immunotolerance mechanism, due to a lack of CSFV recognition by the immature immune system of the fetus and newborn. They will be apparently healthy without an antibody response against CSFV but with high virus replication during their lifetime [3].

In September 2018, CSF reemerged in Japan 26 years after the last CSF case. The first CSF outbreak was reported in domestic pigs in Gifu prefecture, in the central area of Japan. Soon after, wild boars infected with CSFV were found in an area near the first case of CSFV detection in pigs. As of the end of January 2021, a total of 3050 wild boars were confirmed to be infected with CSFV in 23 prefectures. The infections were caused by a moderately virulent CSFV strain genetically belonging to genotype 2.1, which is closely related to recent CSFV strains isolated in China (2015) and Korea (2017–2019) [5,6,7]. Overall, the detection of infected host animals was delayed due to the CSFV infections being caused by moderately virulent strains, which resulted in the presence of asymptomatic clinical signs and silent infection in the susceptible hosts, playing a potential role in the CSF spread in the population. Since the CSF cases were confirmed in wild boars, control measures were implemented such as setting up fencing to restrict wild boar movement, increasing the capturing of wild boars, and disinfection of affected areas wherein wild boars were found as captured and dead, as wild boars are natural reservoirs. Oral vaccination of wild boar was applied to control the CSF spread, starting at the end of March 2019. Our study was conducted first by four vaccinations in two seasons which were implemented continuously with an interval of several weeks until August 2019. The initial CSF outbreaks led to a situation in which CSFV infections in wild boars were continuously detected, and the affected areas of wild boars were expanded.

The surveillance and implementation of oral vaccination were applied to control CSFV infection in wild boar populations; however, the spread of CSF among the wild boar population was expanded to the neighboring prefectures; the effect of oral vaccination in wild boars was unclear. Therefore, our study aimed to describe a vaccine efficacy against CSFV among the wild boar population. On the assessment procedure, disease diversities of wild boars based on virological assessments were assumed according to the antigen and antibody status in each individual to clarify the wild boar population more. This clarification would enable to visualize the disease dynamics of CSFV infections based on the epidemiological analysis. The obtained results can help to understand the role of wild boar in CSFV infections during circulating in the population and improve CSF control measures in wild boars.

## 2. Materials and Methods

### 2.1. Wild Boar Data and Data Sources

Following the confirmation of the initial CSF outbreaks in Gifu prefecture, the investigation of CSF in wild boars began. Hunters and local people who found captured and dead wild boars sent the carcasses to the Livestock Hygiene Service Centers in each area. Under this large-scale surveillance, local veterinarians collected samples from a total of 1443 wild boars between September 2018 and August 2019, and the biological information of sampled wild boars was recorded. The ages of wild boars were estimated according to the body length of the individual wild boar; wild boar with the body length of less than 80 cm, between 80 and 100 cm, and more than 100 cm, were categorized as piglet of less than 1 year old, young wild boar of 1–2 years old, adult wild boar of more than 2 years old, respectively [8]. According to the criteria, 540 individuals were less than 1 year old, 360 individuals were 1–2 years old, and 534 individuals were more than 2 years old; however, nine individuals were not categorized as any of the three. A total of 754 (52.3%) and 682 (47.3%) wild boars were classified as male and female, respectively (Table 1). Sixteen wild boars with unknown information about age or/and gender were not classified into any of the categories.

The corresponding dates and location names of wild boars for epidemiological analysis are provided in Table S1. The geographical coordinates (latitude and longitude) of each wild boar were described through the Geocoding search system [9]. Geographical information about the dissemination of vaccine baits in the prefecture was used to investigate vaccine efficacy in wild boars.

### 2.2. Wild Boar Samples

All wild boar samples of Gifu prefecture were collected under the national surveillance program authorized by the Ministry of the Environment and Ministry of Agriculture, Forestry, and Fisheries (MAFF), Japan. Between September 2018 and August 2019, blood, body fluids (ascites, heartwater, and pleural effusion), and tissue samples (tonsil, spleen, and kidneys) were collected (Table S1). Using these wild boar samples, prefectural Livestock Hygiene Service Centers have conducted the gene-based detection of CSFV using conventional reverse transcription-polymerase chain reaction (RT-cPCR) according to the standard method [10] and antibody detection using enzyme-linked immunosorbent assay (ELISA) [11], then results were reported to the central government. According to results obtained using tonsil samples, 486 individuals (38%) out of 1278 captured wild boars were positive for CSFV by RT-cPCR, and 132 individuals (80%) out of 165 dead wild boars were positive for CSFV by RT-cPCR. From ELISA results, 448 of the captured wild boars (35%) and 51 of the dead wild boars (31%) were antibody-positive (Table S1). Further quantitative analyses described below were conducted under the Agreement of “Chu-oh Kaho No. 18–21” between the Livestock Hygiene Service Centers and Hokkaido University, which was issued on 15 October 2019, as secondary usage of the samples. Besides that, serum samples collected from other 77 wild boars in Mie prefecture were used for evaluation of real-time reverse transcription-polymerase chain reaction (RT-qPCR) and RT-cPCR (Table S2).

### 2.3. RT-qPCR

Viral RNA was extracted from a 50 µL serum or body fluid sample using a MagMax™-96 AI/ND Viral RNA isolation kit (Applied Biosystems, Thermo Fisher Scientific). RT-qPCR reactions were carried out in 20 µL volumes to amplify the 5’ UTR of CSFV using the gene-specific primers CSF 100F and 192R, and the CSF Probe 1 [12]. In the reaction, 2 µL of RNA template was used, and RT-qPCR was carried out with a One Step PrimeScript RT-PCR Kit (Perfect Real Time) (TaKaRa Bio, Shiga, Japan) according to the manufacturer’s instructions. The temperature profile was 5 min at 42 °C and 10 s at 95 °C (reverse transcription), followed by 45 cycles of 15 s at 95 °C, 30 s at 58 °C, and 30 s at 72 °C (PCR amplification). The fluorescence was detected during the annealing step in a LightCycler® 480 instrument.

### 2.4. Luciferase-Based SNT

All serum and body fluid samples were diluted with phosphate-buffered saline-Tween 20 (final concentration, 0.15%). This mixture was treated by heating at 56 °C for 30 min [13]. Serum neutralization test (SNT) using a luciferase-based recombinant virus was performed according to a protocol previously described [14]. The neutralization titers of antibodies against vCSFV GPE^–^/HiBiT were calculated according to luciferase activity measured in the culture supernatants using Nano-Glo HiBiT lytic detection system (Promega, Madison, WI, USA). A cutoff value for the SNT was calculated based on the average number of the mock-infected 96-well plates having five times the standard deviation of tested wild boar sera. The neutralizing antibody titer of each serum was expressed as the highest serum dilution showing complete neutralization of vCSFV GPE^–^/HiBiT.

### 2.5. Regression Analysis of Antibody Detection

As SNT is the standard method to quantify neutralizing titers of antibodies against CSFV infection, sample-to-positive (S/P) values of ELISA were compared with titers of SNT for the determination of the correlation of these values. The analysis was performed using the linear regression method in R [15].

### 2.6. Temporal Trend Analysis of Antibody Responses in Wild Boars Population

The commercial bait vaccine (Pestiporc Oral, IDT Biologika GmbH, Dessau-Rosslau, Germany), an attenuated CSF vaccine based on C-strain was applied to control CSF spread since March 2019 [16]. Vaccine baits were disseminated twice per season in the designed areas according to the guidance of MAFF [17]. The study period involved two seasons with four vaccinations: 1st vaccination, 24–29 March; 2nd vaccination, 7–11 May; 3rd vaccination 10–16 June; and 4th 20–24 August in 2019. Based on the quantitative detection of antibodies against CSFV, the neutralizing antibody titers in wild boar sera were analyzed weekly in a temporal trend analysis according to the calculation of a geometric mean titer (GMT). Also, the proportion of seropositive animals per animals was expressed to show the dynamics of estimated diverse groups and immune responses of wild boars on a weekly base. The geographical analysis was conducted by overlaying the map of oral vaccine dissemination to the locations of wild boar captured or found as dead, which were categorized as vaccinated, to assess the geographical efficacy of bait vaccine dissemination. For the analysis, an area with a 2.5 km diameter was set up and recognized as the vaccinated area associated with the habitat area of wild boars in the field [18]. 

### 2.7. Classification of Quantitative Values

Based on the quantitative values obtained from RT-qPCR and SNT, the investigated wild boars in the present study were classified speculatively into groups using the criteria involving the combination of the degrees of the presence of CSFV and antibody. Critical ranges of the value were applied to the criteria under the classification of individual group and had a different cycle threshold (Ct) value of RT-qPCR and SNT titer. The Ct value of 40 and SNT titer of 1:8 were fixed as the threshold of CSFV-gene detection and antibody detection, respectively. With the quantitative analysis of values, Groups A, B, C, D, and E were described among the wild boar population, and the value ranges were expressed respectively regarding the quantitative classification: Ct values less than 30 and SNT titers less than 8, Group A; Ct values less than 30 and SNT titers between 8 and 32, Group B; Ct values between 30 and 40 and SNT titers equal or less than 128, Group C; Ct values equal or more than 30 and SNT titers more than 128, Group D; Ct values more than 40 and SNT titers between 8 and 128, Group E; and Ct values equal or more than 40 and SNT titers less than 8, no infection.

### 2.8. Multi-Distance Spatial Cluster Analysis and Kernel Density Analysis

To calculate the maximum distance of wild boars classified in the groups, a multi-distance spatial cluster analysis tool in ArcGIS software version 10.6.1 (ESRI, Redlands, CA, USA) was used according to the guidelines provided on the manufacturer’s website and the previous studies [16,19,20]. A common transformation of Ripley’s K function was used in the analysis. For an analysis of the spatial pattern of the group, observed K values were compared to the Expected K values of a completely random spatial distribution of CSF detection with 999 simulations, which is equal to a confidence level of 99.9%. The Diff K values contain the Observed K values minus the Expected K values. The Expected K values producing the highest Diff K values were set as the maximum distance for the relationship between CSF detections in wild boars in Gifu prefecture. Kernel density, a nonparametric estimator for simplifying the spatial level of consequent events, especially for Groups A and B, was estimated using the kernel density tool of ArcGIS 10.6.1. From the processing of Ripley’s function, radiuses of 21.5 km and 11 km were obtained from the calculation for Group A and Group B, respectively. These radiuses were applied as the maximum distance for a significant spatial relationship between CSF detections.

## 3. Results

### 3.1. Quantitative Detection of Viral RNA in Wild Boar Serum

A total of 1443 serum and body fluid samples were collected between September 2018 and August 2019 under large-scale surveillance in Gifu prefecture (Table 1 and Table S1). At the prefectural level, tonsil samples were selected and tested for regular CSF diagnosis according to the standard method approved by the MAFF, Japan. Tonsil samples of 1443 wild boars provided the status of the presence or absence of specific genes of CSFV using RT-cPCR. In our study, due to the limitation of a homogenate of tonsil samples, viral RNA was extracted from the serum or body fluid sample of wild boars and was quantified by RT-qPCR using CSF specific primers and a probe. A Ct value of 40 was applied for the cutoff to distinguish CSFV positive and CSFV negative. Moreover, after the evaluation of RT-qPCR using wild boar serum, the result indicated that RT-qPCR with the cutoff had high sensitivity and specificity at 100% and 98.4%, respectively (Table S2). 

### 3.2. Detection of Antibodies against CSFV

All of the 1443 sera and body fluids were used for the detection of antibodies against CSFV using ELISA in Livestock Hygiene Service Centers of Gifu prefecture to screen the antibody response. To confirm the results, these wild boar samples were further investigated for the quantification of neutralizing antibody titers against CSFV by SNT. As SNT is the golden standard method, the initial dilution of the sample was 1:2, and the presence of antibody response would be judged by this criterion. However, with certain factors such as high toxicity of serum samples in the cell culture, SNT had a 2-fold serial dilution initiated from 1:8. Based on the neutralization antibody titers quantified by SNT, the S/P values of ELISA were compared for a conformability of the tests (Figure S1). The correlation coefficient between the average of S/P values and titers of SNT was calculated as 0.81.

### 3.3. Temporal Trend Analysis of Antibody Response in Wild Boar during With- and Without-Vaccination Period

**Figure 1:**
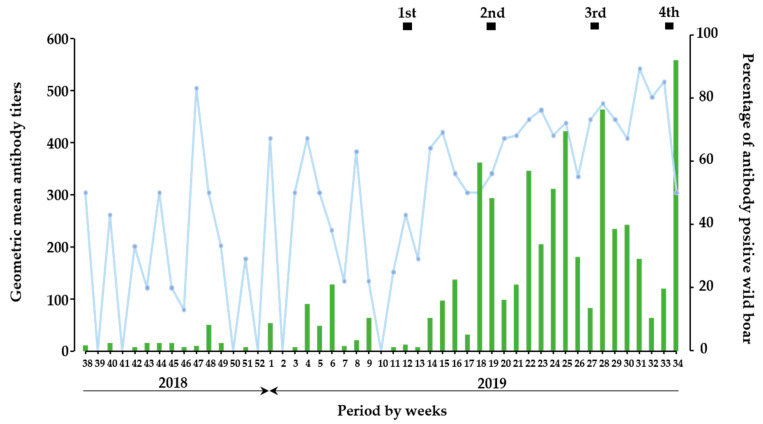
Prevalence of antibodies against CSFV in wild boar. In temporal trend analysis, results on wild boar samples (n = 1166) were combined and analyzed as a week base. The green bar indicates the GMT of SNT in wild boar sera per week, and the blue line indicates the percentage of antibody-positive wild boars in each week. The black box indicates the duration of oral vaccination.

In the temporal analysis, the period of the present study was divided into two parts: before (from September 2018 to February 2019) and after (from March 2019 to August 2019) oral vaccination (Figure 1), and a total of 598 and 568 wild boars were involved in each part, respectively. In context, among 1166 wild boar samples, 8.5% (*n* = 51) and 48% (*n* = 271) antibody-positive wild boars were detected before and after oral vaccination, respectively (Table S3). Before oral vaccination implementation, wild boars developed low neutralizing antibody titers as a GMT of SNT up to 90. On the other hand, SNT titers dramatically increased from the 1st vaccination and further implementation of oral vaccinations resulted in high GMT as approximately 560 SNT titers. Moreover, induction of neutralizing antibody, which observed in each vaccination period was likely to be enhanced by the vaccination; 37 of GMT between the 1st and 2nd vaccination, 57 of GMT between the 2nd and 3rd vaccination, 24 of GMT between the 3rd and 4th vaccination, 90 of GMT in the 4th vaccination. In the context, the proportion of antibody-positive wild boar after initial vaccination indicated that the rate of wild boars with antibody response among the total population per week was kept as a high rate.

### 3.4. Divergent Quantitative Classifications among Wild Boar Population in Gifu Prefecture

In the present study, the quantitative analysis of the detection of viral genes and antibodies against CSFV was applied to clarify the details of the immunized or diseased status of wild boars. For the quantitative analysis of CSFV detection, 1166 out of 1443 wild boar samples were selected regarding the volume of samples, and viral RNAs were quantified by RT-qPCR targeting the 5’UTR region of CSFV [12]. In the test, 282 samples were diagnosed as CSFV positive, and 884 samples were diagnosed as CSFV negative (Table S3). These results were unmatched with the result of RT-cPCR using tonsil sample, which should be due to the difference of sample type (Tables S2 and S4). Ct value of 40 and a neutralizing antibody titer of 1:8 were fixed as the cutoff values to distinguish quantitative values for the analysis. Additionally, value ranges were applied regarding quantitative classifications (Table 2). According to the criteria, approximately 14% of wild boars (*n* = 168) were classified to Group A. About 2% (*n* = 23) of the population was classified to Group B. In addition, the proportions of wild boars in Group C and D were approximately 7% (*n* = 81) and 16% (*n* = 187), respectively.

### 3.5. Proportional Distribution of Diversity of Wild Boars

**Figure 2:**
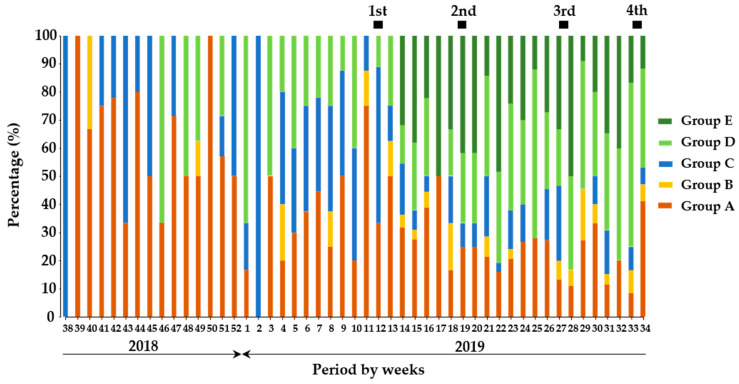
The proportion of wild boars based on the degree of antigen and antibodies against CSFV. In the temporal trend analysis, the results of quantitative analysis on wild boar samples (n = 1166) were combined and analyzed weekly. The dark green, green, blue, orange, and red colors indicate the classifications, Groups A, B, C, D, and E in the wild boar population, respectively. The black box indicates the duration of oral vaccination.

The comparison of proportions among the groups of wild boars indicated that wild boars in Group A dominantly presented in the period before vaccination but decreased to a certain level after the implementation of the initial vaccination (Figure 2). On the other hand, Group B was observed occasionally before vaccination but periodically observed in the vaccination period. Proportions of Group A and B were 11.3% and 0.7%, respectively before vaccination, and slightly increased to 17.4% and 3.0%, respectively after vaccination. The proportion of Group C was still stable for both the before- and after-vaccination periods. Moreover, the proportion of wild boars classified in Group D slightly increased for all periods of after-vaccination compared with before-vaccination. The proportion of wild boars classified in Group E was apparently higher in the periods of after-vaccination compared with ones of before-vaccination.

### 3.6. Efficacy of Oral Vaccine for Wild Boar

**Figure 3:**
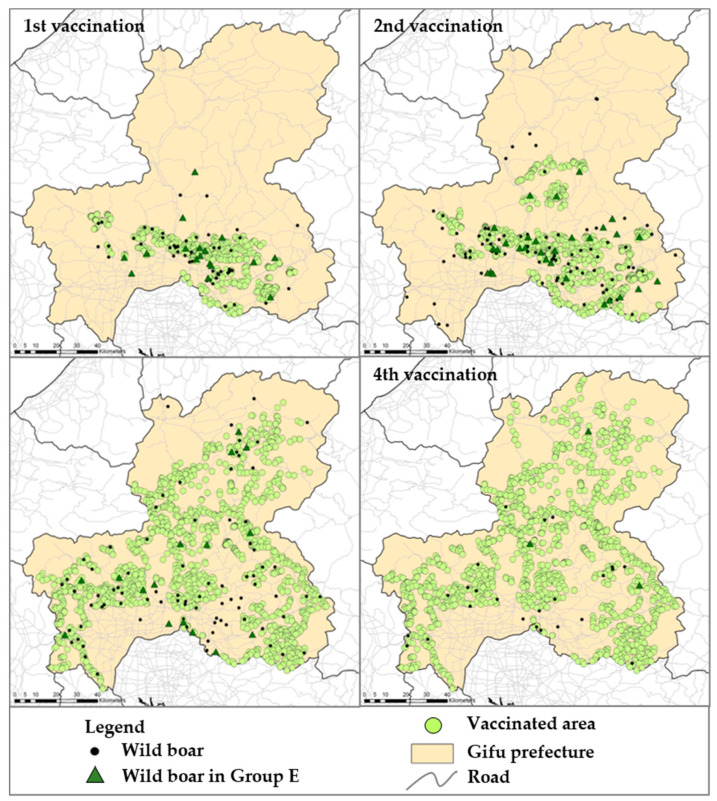
Geographical dissemination of bait vaccine and its response in wild boar. The study period was divided into four regarding each vaccination to visualize the oral vaccination period and the coverage of the vaccination. On the map, black dots indicate the location of the sampled wild boar. The dark green triangles indicate the location where the wild boars in Group E were found in the quantitative analysis. The individual location of dissemination of vaccine baits was indicated by light green circle and the area has been designated as a wild boar habitat zone with an approximately 2.5 km radius.

According to the data source, bait vaccines were disseminated in the surrounding areas of the urban region where most of the wild boars were found with CSFV until February 2019 (Figure S2) and were periodically disseminated more widely (Figure 3). After the implementation of the first vaccination, the number of antibody-positive wild boar increased, and the development of neutralizing antibody titers progressively increased to approximately 560 of GMT (Figure 1). In the analysis, 83 (7%) wild boars were classified in Group E, implying potential wild boars with vaccine response, likely to be protected against CSFV infection overall vaccination periods. However, five out of these wild boars were found to be dead in a field, which could have been caused by other factors such as hunger. The numbers of wild boars with potentially protective immunity against CSFV infection were 26, 35, 19, and 3 out of 568 wild boars in the 1st, 2nd, 3rd, and 4th vaccination periods, respectively. By age group, 15, 27, and 41 wild boars were categorized as vaccine response with age ranges of less than 1 year old, 1–2 years old, and more than 2 years old, respectively (Table 2). Moreover, the geographical analysis showed that 14 wild boars (17%) were found outside of the oral vaccine disseminated area, throughout the entire vaccination periods.

In addition, wild boars classified in Group A were dominantly observed as 27% among total wild boars in the 1st vaccination period, and the other groups were low proportion between 3 and 23%. Between the 2nd and 4th vaccination periods, the dominant wild boar group was Group D, having an apparent increase as ranged between 28 and 34%. Moreover, the proportion of wild boars in Group A periodically decreased until the 4th vaccination, having the range between 10 and 19%, and this trend was also observed in wild boars in Groups B and C throughout the periods. Taken together, it was suggested that continuous vaccination events contributed to the induction of protective immunity among wild boar population. 

### 3.7. Kernel Density Estimation Analysis

The kernel density estimation was applied to wild boar in Groups A and B to visualize the spatial distribution of wild boars. It was indicated that wild boars in Group A were initially found in the southern part of the prefecture with a high density and were eventually disseminated in the entire prefecture (Figure S3). In Group A (*n* = 168), 74% of wild boars were found in areas at a high density (Level 4–6), and 16% were found in areas with low density (Level 1–3). The analysis on wild boars classified in Group B indicated that a total of 23 wild boars were located in several areas that had several hot spots with variable density; 70% of them were located in six areas with higher density (Level 3–4), and 30% of wild boars were located in five areas with lower density (Level 1–2) (Figure S4).

**Figure 4:**
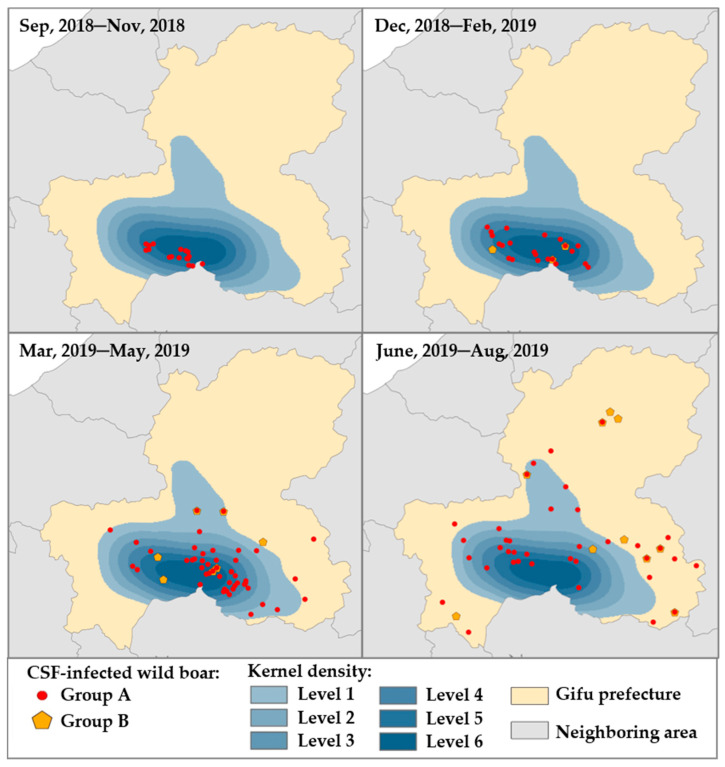
Dynamics of two groups of wild boars classified as a potential natural reservoir of CSFV among wild boar population. The illustration of Groups A and B was arranged for 4-time courses, as shown on the map. The heat map indicated the estimated kernel density for Group A (red circle) as one epicenter from high density (dark blue, level 4–6) to low density (light blue, level 1–3). Group B (yellow pentagon) was illustrated in the geographical map for the same period.

To investigate the relationship between Groups A and B with time sequence in terms of CSFV infection, the occurrence of CSFV infections in both groups by every three-month period was overlaid with the strata map of the kernel density analysis (Figure 4). Recognition of wild boars in Group A was limited only in high-density areas, but there were none of the wild boars in Group B during the first period (between September 2018 and November 2018). In the second (between December 2018 and February 2019) and third periods (March 2019 and May 2019), wild boars in Groups A and B existed in the areas with high density and had been gradually disseminated within the equilibrium. Between June 2019 and August 2019, both groups were widely scattered throughout the entire prefecture, and more than half of them were found even outside of high density areas.

## 4. Discussion

The last notification of a CSF case in Japan was in 1992, and a CSF-free declaration was recognized by OIE in 2007. Based on the unique feature of the country having no bordering countries, the implementation of control measures for disease prevention in Japan is not complicated and without concern of animal movement through the bordering area illegally. As moderately virulent CSFV emerged in wild boars in Gifu prefecture, large-scale surveillance has been implemented since September 2018, and oral vaccination was applied from March 2019 to the present in Japan. The dynamics and risk factors of CSF in wild boars in Japan had been already indicated based on epidemiological data [16,20]. In the present study, the proportions of antibody-positive wild boars and GMT of SNT were indicated to describe immune status. In addition, we demonstrated new approaches that RT-qPCR and SNT can provide more precise and quantitative information about the development of immune response and CSFV circulation among the host population to obtain an accurate overview of disease spread in wild boars. 

In the present study, the study period involved a time with natural infection of CSFV and with the implementation of oral vaccination. The quantitative analysis revealed that the proportion of antibody-positive wild boars increased continuously during the vaccination period compared with the before-vaccination period, and approximately 48% of wild boars would cause the induction of antibody response in the vaccination periods. The increase of antibody-positive rate in the affected area might be attributed to a background of the immune status of wild boar population during natural infection of CSFV between 2018 and 2019. A similar observation is also mentioned in the previous study [21].

As recommended by OIE, tonsil is the best sample for the confirmatory diagnosis of CSF [22]. In the present study, the viral gene in serum and body fluids were quantified by RT-qPCR regarding the ability of the samples from wild boar. Furthermore, quantitative detections of antigen and antibodies against CSFV among the population allow understanding the disease characters, the effectiveness of the vaccine, and progressive changes [11,23]. With this reason, our quantitative analysis involving high-throughput methods suggested that the wild boar population has several characteristics in the infection. The quantitative findings demonstrated that there were wild boar groups with different backgrounds of the amounts of the viral gene and immune response, which may illustrate the diversity of “CSFV infection form” depending on the virulence of the field strain and host factors [2,24,25]. These variable backgrounds were demonstrated in the animal experiment using pigs and field vaccine study in wild boars [21,26]. The groups are likely to be related with CSFV infections; Groups A, B, C, D, and E are presumed to acute or persistent infection, chronic infection, subclinical infection, recovery, and vaccine response, respectively. Moreover, the CSFV infection caused by moderately virulent strains resulted in multi infection forms in host animals regarding the difference of age and health condition of wild boar. Among them, wild boars in Groups A and B are assumed to carry out the major characteristic of disease transmission, and these wild boars were potential carriers contributing to the disease maintenance in the field, which was pointed out by European studies [25,27]. As studies described, the risk of carrier pigs with acute or persistent infection, and chronic infection were reported, which highlighted that direct and indirect contacts within the susceptible host could contribute to increasing the risk of CSFV infection and viral transmission among wild boars and domestic pigs [28,29]. 

Based on these findings of the quantitative detection of antigen and antibody, the temporal analysis of CSFV infections revealed that, in before vaccination, the major infection was assumed acute or persistent infections, and chronic infection (Figure 2). In this assumption, the proportion of wild boars with acute or persistent infection was dominant in this period but decreased by a certain level during vaccination. Interestingly, wild boar with likely chronic infection would be confirmed occasionally before vaccination and was periodically observed in the vaccination period. This would be explained by the evidence that in the vaccinated period, under the assumption that the vaccine response was induced after the implementation of oral vaccination, the profile of groups changed their proportions and the number of antibody-positive wild boars with high SNT titers increased (Figure 1 and Figure 2). On the other hand, the epidemiological analysis suggested that occurrences of potential chronic infection would be overlapped with occurrences of acute or persistent infection in wild boar in the field. It would lead to maintaining of CSFV in the population through long-term infection (Figures S3 and S4). It means that once the infections occurred widely, the difficulty of CSF control in wild boar was demonstrated similar to previous results [25].

The quantitative approach would describe wild boar status in terms of the vaccination in the CSF affected area. For the efficacy of the oral vaccine, the vaccine response was estimated in 7% of wild boars in the population. Among them, the small proportion of piglets with antibody response indicated that the uptake of the oral vaccine by piglets would be limited. This phenomenon is also indicated in the study of Rossi et al. [27]. That is why we need to implement long-term vaccination to cover all ages of wild boar, including a new generation of the herd, to prevent vertical transmission. In detail, antibody responses, including vaccine response and recovery status, were assumed in wild boar. After each oral vaccination, wild boars with an antibody response induced by the oral vaccine were obtained in a proportional range of between 40% and 50% in weekly time points, describing the maintenance of the antibody-positive population in CSF affected areas. This statement demonstrates that the efficacy of the oral vaccine in wild boar supports the results of experimental studies of oral vaccines [21,30]. In terms of antibody response, wild boars assumed as being at a recovery status increased apparently after vaccination from 9% to 26%. Collectively, the increase of the numbers of wild boars with antibody response was likely to be a positive impact of oral vaccination and decrease of CSFV infections in the affected area [31]. However, the limitation is that a clear description of the role of an oral vaccine and the recovery status in terms of CSFV infection was not available in the present study; recent vaccine studies have focused on reliable differentiation of CSF-infected from the vaccinated host [21,32].

Using the criteria based on the quantitative assessment, the disease dynamics (Figure 4) and distribution of wild boars during the vaccination period (Figure 3) suggested that the density of the wild boar population in areas and habitation of wild boar strongly contributed to CSFV spread in the field; these results support previous findings [25]. In our analysis, an area with a 2.5 km diameter was set as the effective area of each bait associated with the habitat area of wild boars in the field [18]. Wild boars with a vaccine response assumed were found in non-vaccinated areas during the oral vaccination period, which may suggest that wild boars should have flexible home ranges of more than a radius of 2.5 km [33]. It was also reported that wild boars are able to shift between habitat types of forest and open areas according to availability of food resources; sows with piglets may change habitat areas because of their sensitivity to human disturbance, and wild boars, especially solitary male wild boars, can have a larger habitat area than females and may re-visit that area at any time [34]. These findings strongly suggested that under a flexible strategy of oral vaccination based on a predictive study, continues vaccinations should contribute to the induction of protective immunity and maintain its level in the population for the long term.

In the quantitative analysis, there is a limitation on the classification of wild boars shown antibody response by either vaccine response or recovery status in CSFV infection. As studies have indicated that depending on virulence of CSFV, the host species would show a variety of clinical signs, however, parallel antibody response is an indicator of progressive CSFV infection or effectiveness of the vaccine in the host [11,23]. To clarify this point, antigen presence and antibody response should be monitored quantitatively at least twice with a time interval.

## 5. Conclusions

In order to assess the vaccine efficacy, the antibody-positive proportion and GMT of wild boars were quantitatively assessed to visualize immune status. RT-qPCR was applied and provided accurate information for large-scale surveillance. CSFV infections as likely acute or persistent infections and chronic infection caused by moderately virulent CSFV play an important role among wild boar population. Oral vaccination for wild boar could contribute to the decrease of CSFV infections in the affected area. The implementation of effective control measures for wild boars, the combination of continuous oral vaccination and monitoring of antigen and antibody response among the population, and depopulation of wild boars are critical to eliminating CSFV infection in wildlife.

## Figures and Tables

**Table 1:** Summary of wild boar samples collected in Gifu prefecture between September 2018 and August 2019.

Age Group	Total	Gender			Status	
		Male	Female	Unknown	Captured	Dead
≤1 year	540	271	266	3	478	62
1–2 years	360	176	181	3	328	32
>2 years	534	302	231	1	463	71
Unknown	9	5	4	0	9	0
Total	1443	754	682	7	1278	165

**Table 2:** Quantitative classification of the antigen and antibodies against CSFV.

Groups	Cutoff Values		Total	Age				Status	
	RT-qPCR	SNT		≤1	1–2	>2	Un- known	Captured	Dead
Group A	<30	<8	168	57	45	66	0	130	38
Group B	<30	8–32	23	11	5	7	0	16	7
Group C	30–40	≤128	81	18	22	41	0	54	27
Group D	≥30	>128	187	24	62	101	0	185	2
Group E	≥40	8–128	83	15	27	41	0	78	5
No infection	≥40	<8	624	329	130	156	9	606	18
Total			1166	454	291	412	9	1069	97

## Data Availability

All data generated or analyzed during this study are including in this article.

## Supplementary Materials

The following are available online at https://www.mdpi.com/1999-4915/13/2/319/s1, Table S1: List of wild boar samples collected from in Gifu prefecture between September 2018─August 2019. Table S2: Comparison of RT-cPCR and RT-qPCR using wild boar sera collected in Mie prefecture, Japan (*n* = 77). Table S3: The summary for results of RT-qPCR and SNT using wild boar samples collected in Gifu prefecture, Japan (*n* = 1166). Table S4: Comparison of RT-cPCR and RT-qPCR using wild boar samples collected in Gifu prefecture, Japan (*n* = 1166). Figure S1: Quantitative association of wild boar serum samples between serum neutralization titers and S/P values of ELISA. Antibody responses of 1443 wild boar sera were expressed as the neutralizing titers of SNT and S/P values of ELISA and were plotted. The value of the correlation coefficient (R^2^ = 0.81; *p* < 0.001) indicated a statistically significant linear correlation. Figure S2: Geographical map of Gifu prefecture. The map indicates the natural structure of the landscape and localization of the urban area in Gifu prefecture. Areas of urban, agriculture, grassland, mixed forest, and mountain and deep forest are highlighted by light gold, light green, green, dark green, and darkest green colors, respectively. A red line highlights the prefectural border between neighboring prefectures. Figure S3: Spatial-time change of wild boar classified in Group A among wild boar population in Gifu prefecture. The heat map illustrates the estimated kernel density of Group A as likely to wild boar with acute or persistent infection of CSF and there was one epicenter from high density (dark blue, level 4–6) to low density (light blue, level 1–3). The highest density of the infection form was recognized in the southern area of Gifu prefecture. The study period was arranged by 4-time courses, red circle, September–November 2018; yellow circle, December 2018–February 2019; light green, March–May 2019; and white circle, June–August 2019. Figure S4: Spatial-time change of wild boar classified in Group B among wild boar population in Gifu prefecture. The map illustrates the estimated kernel density of Group B as likely to wild boar with chronic infection of CSF and the analysis indicated that it had several hot spots, from high density (dark orange, level 4–6) to low density (light orange, level 1–2). The study period was arranged by 3-time courses, yellow pentagon, December 2018–February 2019; light green pentagon, March–May 2019; and white pentagon, June–August 2019.
