# Supplementary material for: Efficacy of Oral Vaccine against Classical Swine Fever in Wild Boar and Estimation of the Disease Dynamics in the Quantitative Approach
Source: Viruses. 2021 Feb 20;13(2):319. doi: 10.3390/v13020319 (PMC7924559; doi:10.3390/v13020319)
Supplement: Supplementary file 1 [file viruses-13-00319-s001.zip › 6. viruses-1078680_20210219-suppl/3. Figure S1-4 20210219.pptx]

## Slide 1
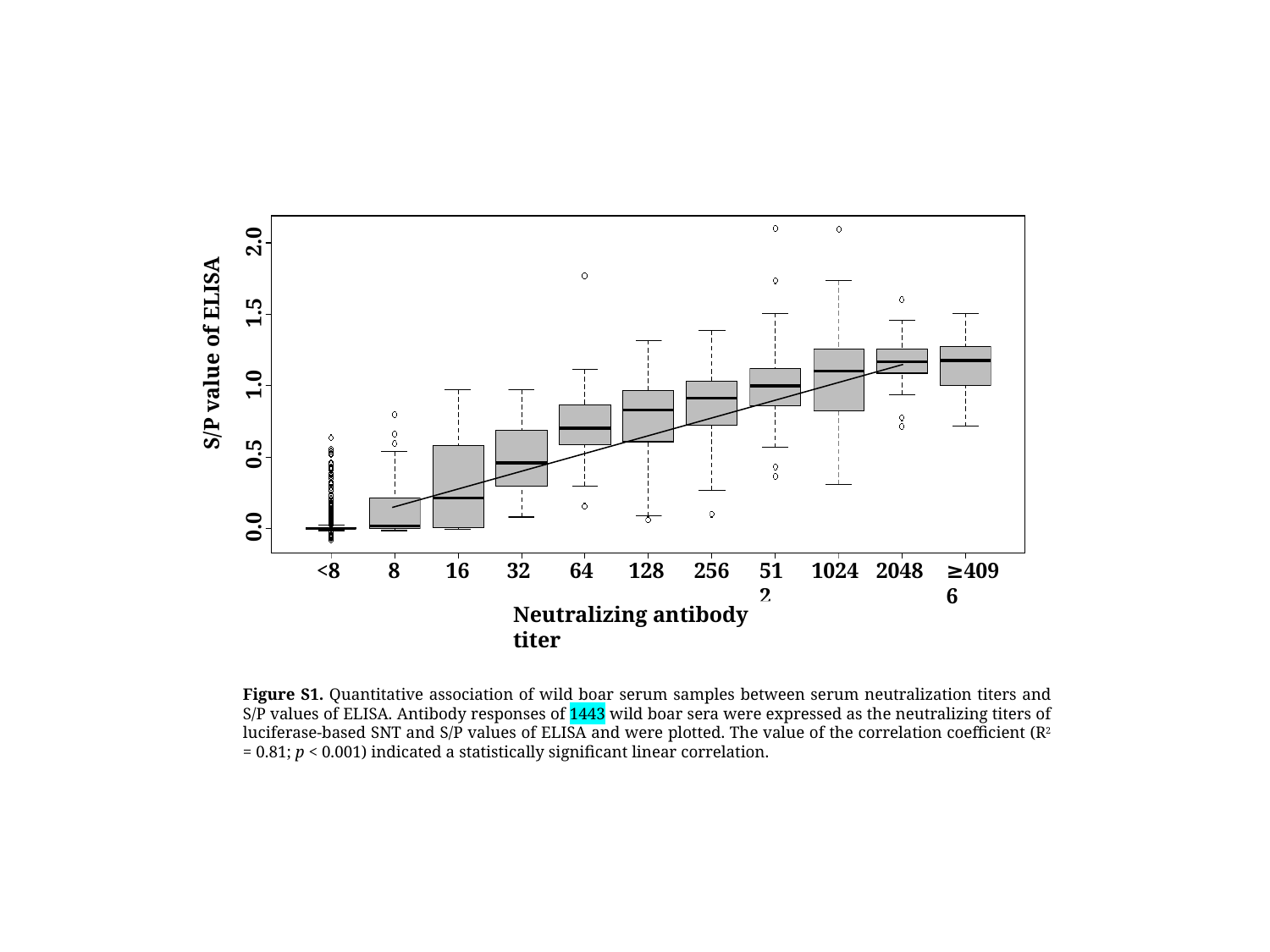

2.0
S/P value of ELISA
1.5
1.0
0.5
0.0
<8
8
16
32
64
128
256
512
1024
2048
≥4096
Neutralizing antibody titer
Figure S1. Quantitative association of wild boar serum samples between serum neutralization titers and S/P values of ELISA. Antibody responses of 1443 wild boar sera were expressed as the neutralizing titers of luciferase-based SNT and S/P values of ELISA and were plotted. The value of the correlation coefficient (R2 = 0.81; p < 0.001) indicated a statistically significant linear correlation.

## Slide 2
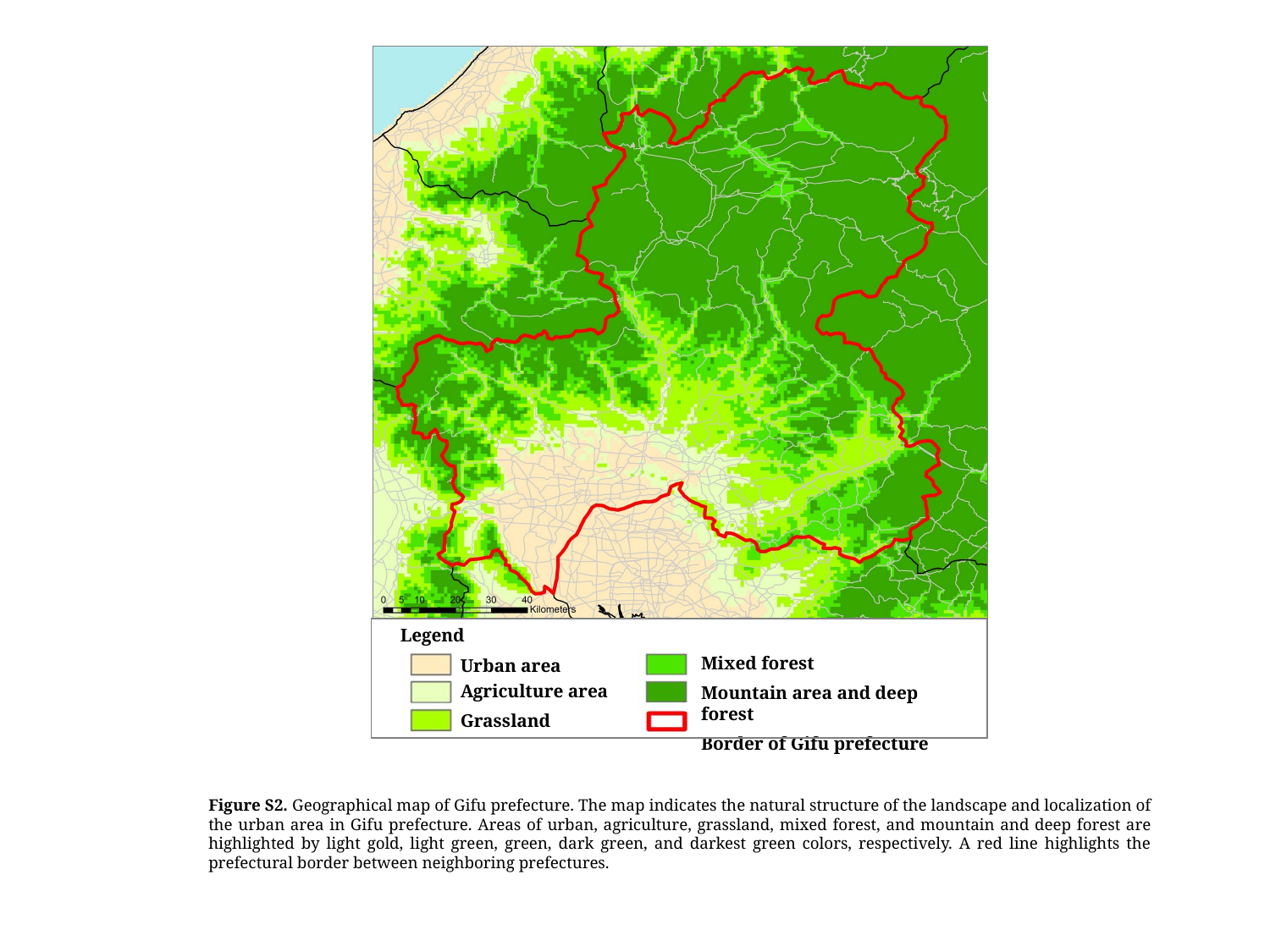

Legend
Mixed forest
Mountain area and deep forest
Border of Gifu prefecture
Urban area
Agriculture area
Grassland
Figure S2. Geographical map of Gifu prefecture. The map indicates the natural structure of the landscape and localization of the urban area in Gifu prefecture. Areas of urban, agriculture, grassland, mixed forest, and mountain and deep forest are highlighted by light gold, light green, green, dark green, and darkest green colors, respectively. A red line highlights the prefectural border between neighboring prefectures.

## Slide 3
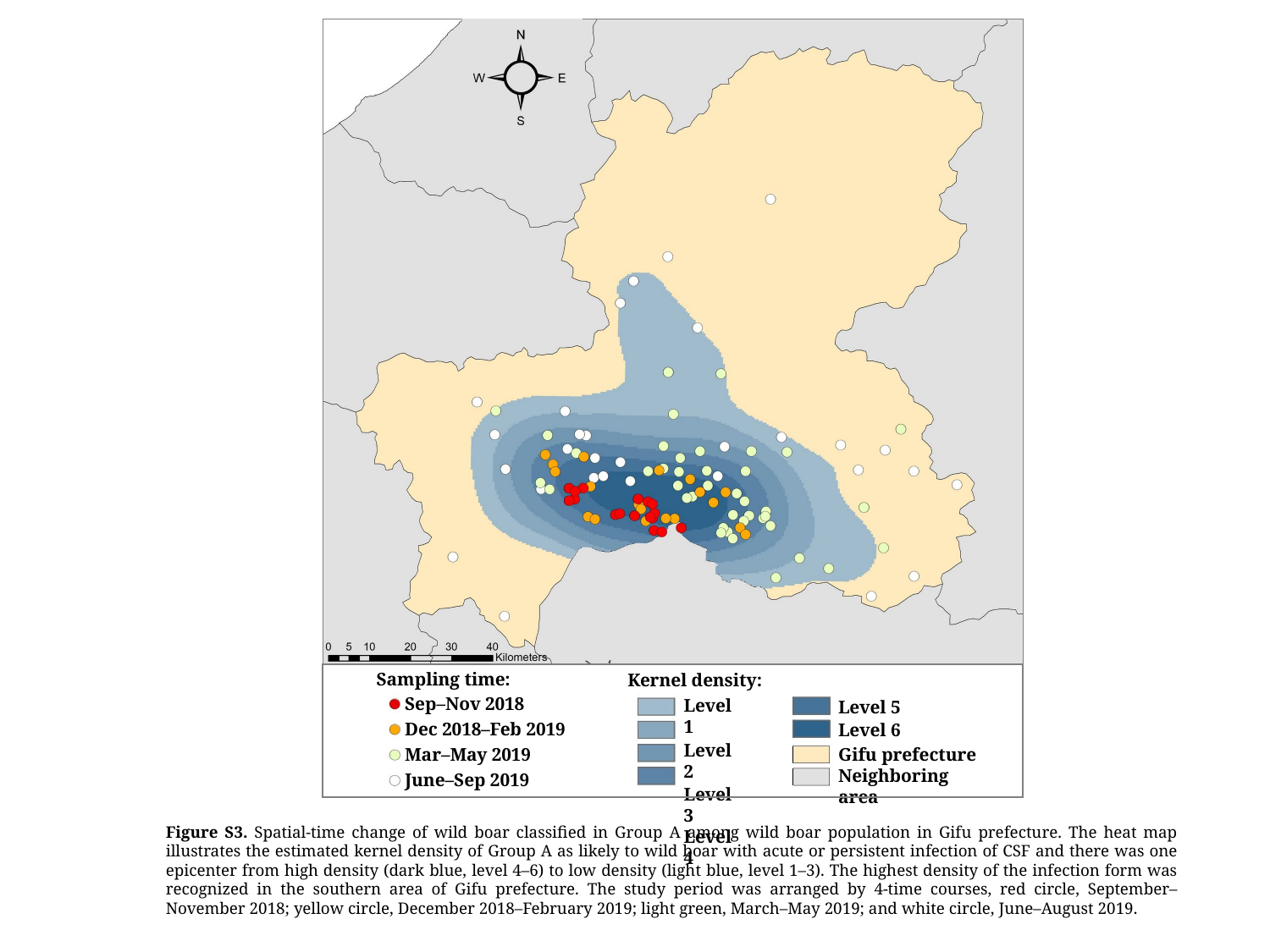

Sampling time:
Sep–Nov 2018
Dec 2018–Feb 2019
Mar–May 2019
June–Sep 2019
Kernel density:
Level 1
Level 2
Level 3
Level 4
Level 5
Level 6
Gifu prefecture
Neighboring area
Figure S3. Spatial-time change of wild boar classified in Group A among wild boar population in Gifu prefecture. The heat map illustrates the estimated kernel density of Group A as likely to wild boar with acute or persistent infection of CSF and there was one epicenter from high density (dark blue, level 4–6) to low density (light blue, level 1–3). The highest density of the infection form was recognized in the southern area of Gifu prefecture. The study period was arranged by 4-time courses, red circle, September–November 2018; yellow circle, December 2018–February 2019; light green, March–May 2019; and white circle, June–August 2019.

## Slide 4
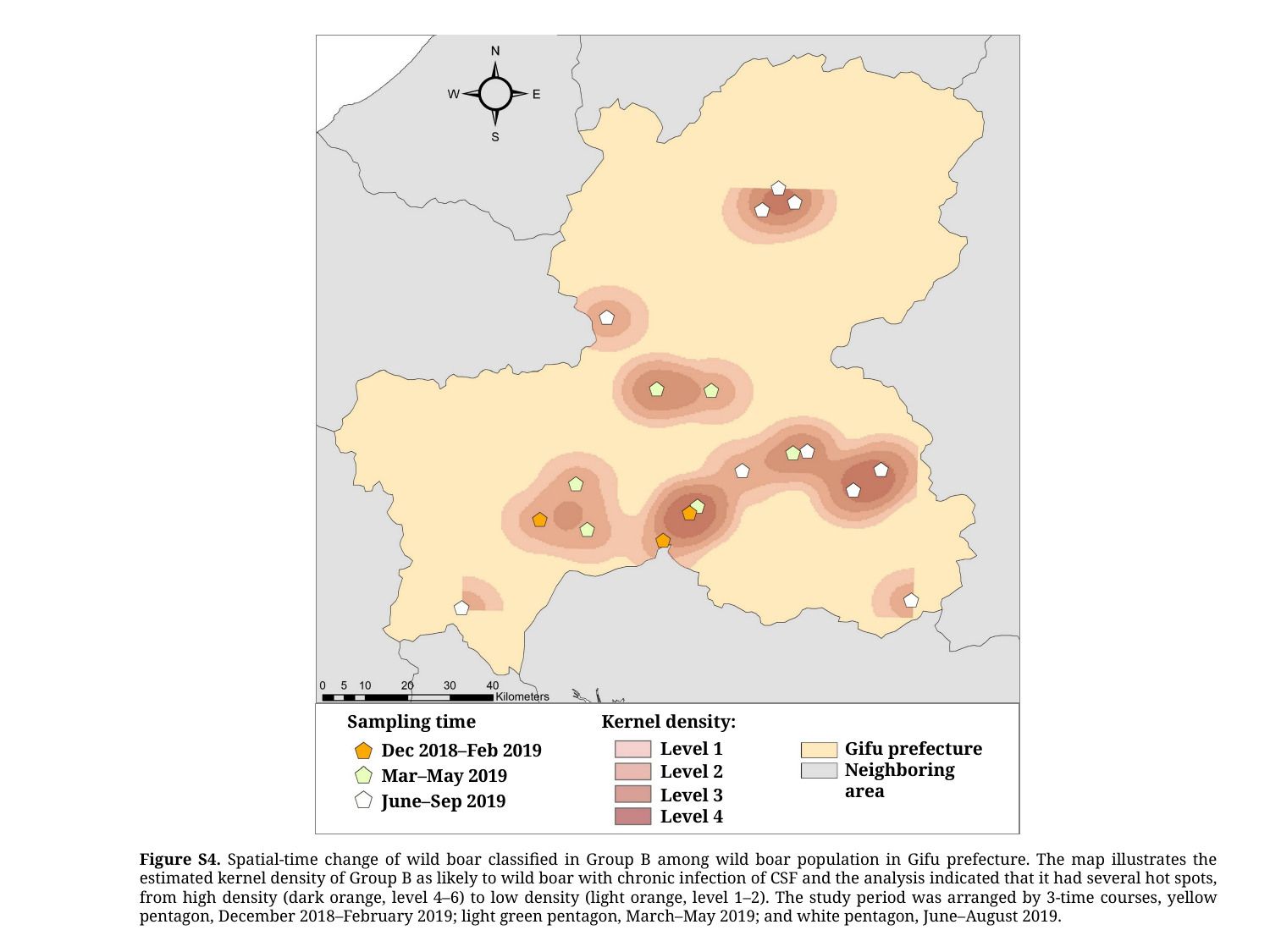

Sampling time
Kernel density:
Level 1
Level 2
Level 3
Level 4
Gifu prefecture
Neighboring area
Dec 2018–Feb 2019
Mar–May 2019
June–Sep 2019
Figure S4. Spatial-time change of wild boar classified in Group B among wild boar population in Gifu prefecture. The map illustrates the estimated kernel density of Group B as likely to wild boar with chronic infection of CSF and the analysis indicated that it had several hot spots, from high density (dark orange, level 4–6) to low density (light orange, level 1–2). The study period was arranged by 3-time courses, yellow pentagon, December 2018–February 2019; light green pentagon, March–May 2019; and white pentagon, June–August 2019.
