# Supplementary material for: Efficacy of Oral Vaccine against Classical Swine Fever in Wild Boar and Estimation of the Disease Dynamics in the Quantitative Approach
Source: Viruses. 2021 Feb 20;13(2):319. doi: 10.3390/v13020319 (PMC7924559; doi:10.3390/v13020319)
Supplement: Supplementary file 1 [file viruses-13-00319-s001.zip › 6. viruses-1078680_20210219-suppl/5. Table S2-4 20210219.docx]

**Table S2.** Comparison of RT-cPCR and RT-qPCR using wild boar sera collected in Mie prefecture, Japan (n = 77)

| **Assays** | | **RT-cPCR** | | Total |
| --- | --- | --- | --- | --- |
|  |  | Positive | Negative |  |
| **RT-qPCR** | Positive | 16 | 1 | 17 |
|  | Negative | 0 | 60 | 60 |
| Total | | 16 | 61 | 77 |

**Table S3.** The summary for results of RT-qPCR and SNT using wild boar samples collected in Gifu prefecture, Japan (n = 1166)

| **Assays** | **Total**  **sample** | **Before-oral vaccination** | | |  |  | **After-oral vaccination** | |
| --- | --- | --- | --- | --- | --- | --- | --- | --- |
|  |  | n^*^ | Positive | Negative |  | n | Positive | Negative |
| RT-qPCR | 1166 | 598 | 110 | 488 |  | 568 | 172 | 396 |
| SNT | 1166 | 598 | 51 | 547 |  | 568 | 271 | 297 |

* : n, the number of tested animals

**Table S4.** Comparison of RT-cPCR and RT-qPCR using wild boar samples collected in Gifu prefecture, Japan (n = 1166)

| **Assays** | | **RT-cPCR**  **(Tonsil)** | | Total |
| --- | --- | --- | --- | --- |
|  |  | Positive | Negative |  |
| **RT-qPCR**  **(Serum)** | Positive | 266 | 16 | 282 |
|  | Negative | 199 | 685 | 884 |
| Total | | 465 | 701 | 1166 |
